# Mechanisms of Caffeine-Induced Inhibition of UVB Carcinogenesis

**DOI:** 10.3389/fonc.2013.00144

**Published:** 2013-06-17

**Authors:** Allan H. Conney, Yao-Ping Lu, You-Rong Lou, Masaoki Kawasumi, Paul Nghiem

**Affiliations:** ^1^Susan Lehman Cullman Laboratory for Cancer Research, Department of Chemical Biology, Ernest Mario School of Pharmacy, Rutgers, The State University of New Jersey, Piscataway, NJ, USA; ^2^Allan H. Conney Laboratory for Anticancer Research, Guangdong University of Technology, Guangzhou, China; ^3^Division of Dermatology, Department of Medicine, University of Washington, Seattle, WA, USA

**Keywords:** sunlight-induced skin cancer, sunscreen, ATR inhibition, upregulation of p53, coffee

## Abstract

Sunlight-induced non-melanoma skin cancer is the most prevalent cancer in the United States with more than two million cases per year. Several studies have shown an inhibitory effect of caffeine administration on UVB-induced skin cancer in mice, and these studies are paralleled by epidemiology studies that indicate an inhibitory effect of coffee drinking on non-melanoma skin cancer in humans. Strikingly, decaffeinated coffee consumption had no such inhibitory effect. Mechanism studies indicate that caffeine has a sunscreen effect that inhibits UVB-induced formation of thymine dimers and sunburn lesions in the epidermis of mice. In addition, caffeine administration has a biological effect that enhances UVB-induced apoptosis thereby enhancing the elimination of damaged precancerous cells, and caffeine administration also enhances apoptosis in tumors. Caffeine administration enhances UVB-induced apoptosis by p53-dependent and p53-independent mechanisms. Exploration of the p53-independent effect indicated that caffeine administration enhanced UVB-induced apoptosis by inhibiting the UVB-induced increase in ATR-mediated formation of phospho-Chk1 (Ser345) and abolishing the UVB-induced decrease in cyclin B1 which resulted in caffeine-induced premature and lethal mitosis in mouse skin. In studies with cultured primary human keratinocytes, inhibition of ATR with siRNA against ATR inhibited Chk1 phosphorylation and enhanced UVB-induced apoptosis. Transgenic mice with decreased epidermal ATR function that were irradiated chronically with UVB had 69% fewer tumors at the end of the study compared with irradiated littermate controls with normal ATR function. These results, which indicate that genetic inhibition of ATR (like pharmacologic inhibition of ATR via caffeine) inhibits UVB-induced carcinogenesis support the concept that ATR-mediated phosphorylation of Chk1 is an important target for caffeine’s inhibitory effect on UVB-induced carcinogenesis.

More than two million cases of non-melanoma skin cancer occur each year in the United States (Siegal et al., 2012), and the number of these cancers has been increasing (Athas et al., 2003; Rogers et al., 2010). Indeed, the number of cases of non-melanoma skin cancer in the United States each year exceeds that for all of the other cancers combined. Accordingly, approaches for preventing these cancers, which are caused predominantly by overexposure to sunlight, are important. The inhibitory effect of caffeine on UVB-induced skin cancer in animal models described in this manuscript is paralleled by epidemiology studies indicating that drinking regular coffee, but not decaffeinated coffee, decreases the risk of non-melanoma skin cancer in humans (Jacobson et al., 1986; Abel et al., 2007; Song et al., 2012).

Several investigators have evaluated the effect of administration of caffeine post-UVC irradiation on the removal of thymine dimers and on the formation of UV-induced mutations in bacteria and cultured mammalian cells. Different results were obtained in different cell systems. In early studies, Sideropoulos and Shankel (1968) demonstrated that caffeine inhibited excision of thymine dimers from an excision repair proficient strain of *E. coli* that had been irradiated with UV, and this inhibitory effect of caffeine on excision repair was associated with increased UV-induced mutations. In *E. coli* strains (Hcr−) that lacked excision repair, post-UV administration of caffeine inhibited UV-induced mutations, and it was suggested that “caffeine could diminish mutagenesis in Hcr− strains by modifying the process of recombinational repair in a way that reduces its inaccuracy” (Witkin and Farquharson, 1969). In Chinese hamster cells lacking appreciable excision repair of thymine dimers, post-UV treatment with caffeine also inhibited UV-induced mutations (Trosko and Chu, 1973) possibly by inhibiting an error-prone repair system. In an additional study with Chinese hamster cells exposed to UV, caffeine inhibited the rescue of stalled replication forks by translesional DNA synthesis thereby causing a switch to bypass via homologous recombination (Johansson et al., 2006). The caffeine-induced decrease in translesional DNA synthesis was associated with decreased UV-induced mutations and with increased homologous recombination that resulted in increased chromosome aberrations (Johansson et al., 2006). Additional studies are needed to determine the effects of caffeine on error-free and error-prone repair of UV damage in bacteria, cultured mammalian cells, and in mice.

In the present manuscript, we describe animal data indicating that caffeine administration inhibits UVB-induced carcinogenesis by functioning as a sunscreen, as well as by enhancing UVB-induced apoptosis in the epidermis of UVB-treated mice by p53-dependent and p53-independent mechanisms. In long-term studies, caffeine administration enhances apoptosis in tumors of mice treated chronically with UVB.

## Early Studies Leading to Our Interest in the Effect of Tea and Caffeine on UVB-Induced Skin Cancer

Studies in our laboratory (AHC) demonstrated a strong inhibitory effect of ellagic acid, quercetin, myricetin, tannic acid, and many other plant phenols on the mutagenic action of the bay-region diol epoxide that is an ultimate carcinogenic metabolite of benzo[a]pyrene (Wood et al., 1982; Huang et al., 1983, 1985). Additional studies demonstrated that the mechanism of inhibition was by formation of a covalent adduct of the diol epoxide with the polyphenol (Sayer et al., 1982). *In vivo* studies indicated that ellagic acid strongly inhibited the tumorigenic activity of the bay-region diol epoxide of benzo[a]pyrene in mice (Chang et al., 1985), but ellagic acid and other polyphenols were only weak inhibitors of the tumorigenic action of benzo[a]pyrene (Chang et al., 1985). Because of our interest in plant phenols as inhibitors of carcinogenesis, we noted studies by Yoshizawa et al. (1987), who demonstrated that epigallocatechin gallate, a major polyphenol in green tea, inhibited tumor promotion on mouse skin. We also noted the studies by Mukhtar and his colleagues indicating an inhibitory effect of a green tea polyphenol fraction on UVB-induced carcinogenesis (Wang et al., 1991). These studies stimulated us to start studies with tea that led to our finding of caffeine as a strong inhibitor of UVB-induced carcinogenesis (Huang et al., 1997).

## Inhibitory Effect of Oral Administration of Caffeine on UVB-Induced Complete Carcinogenesis

Our interest in caffeine was enhanced after finding that although oral administration of green and black tea inhibited UVB-induced complete carcinogenesis in SKH-1 mice, the decaffeinated teas were inactive, and high dose levels of the decaffeinated teas actually increased UVB-induced carcinogenesis (Huang et al., 1997). Adding back caffeine to the decaffeinated teas restored their inhibitory effects on UVB-induced carcinogenesis, and administration of caffeine alone had a strong inhibitory effect (Huang et al., 1997). The effects of oral administration of green tea, decaffeinated green tea, decaffeinated green tea plus caffeine, and caffeine alone on UVB-induced complete carcinogenesis are shown in Table 1. The results indicated that oral caffeine during the course of twice a week exposure to UVB for 40 weeks and for an additional 4 weeks post-UVB strongly inhibited tumorigenesis. Shortly after we found an inhibitory effect of oral caffeine on UVB-induced carcinogenesis, we became aware of an earlier report indicating that topical application of caffeine just prior to each irradiation with UVB inhibited UVB-induced carcinogenesis in mice (Zajdela and Latarjet, 1978). Since UVA and solar radiation (UVA + UVB) are tumorigenic in mice (de Laat et al., 1997; Li et al., 2012), additional studies are needed to determine the effects of caffeine on tumorigenesis in animals exposed to UVA or solar radiation.

**Table 1:** **Effect of green tea and decaffeinated green tea on UVB-induced complete carcinogenesis**.

Treatment	Number of keratoacanthomas per mouse	Number of squamous cell carcinomas per mouse
Water	5.75 ± 1.04	1.17 ± 0.27
Green tea	2.21 ± 0.46*	0.52 ± 0.18*
Decaf. green tea	4.58 ± 0.64	1.35 ± 0.29
Caffeine	1.81 ± 0.44*	0.63 ± 0.14*
Decaf. green tea + caffeine	2.53 ± 0.43*	0.47 ± 0.11*

Female SKH-1 mice were treated with UVB (30 mJ/cm^2^) twice weekly for 40 weeks. Tea leaf extracts (1.25 gm tea leaf/100 ml hot water; 4 mg tea solids/ml) or caffeine (0.36 mg/ml) were administered as the drinking fluid during the entire period of UVB irradiation and for an additional 4 weeks. Each value is the mean ± SE from 24 to 30 mice. *P < 0.05. Taken from Huang et al. (1997).

## Sunscreen Effect of Caffeine and Caffeine Sodium Benzoate

**Figure 1:**
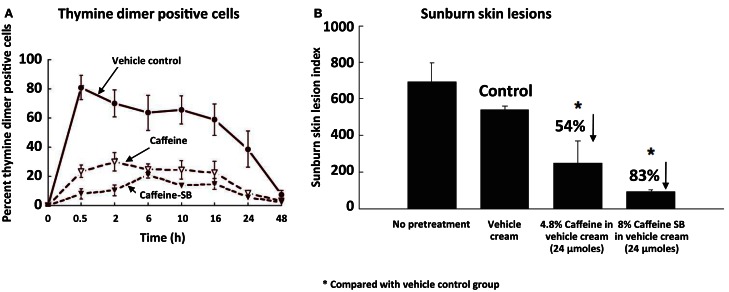
**Inhibitory effect of topical application of caffeine or caffeine sodium benzoate (caffeine-SB) prior to UVB irradiation on UVB-induced formation of thymine dimers and sunburn lesions in the epidermis of SKH-1 mice**. In **(A)**, female SKH-1 mice (7–8 weeks old; five per group) were untreated or treated topically with 100 mg of vehicle cream (Dermabase cream), or with caffeine or caffeine-SB (24 μmol) in vehicle cream 0.5 h before a single irradiation with UVB (180 mJ/cm^2^). The animals were killed just before UVB (“0 h”) or at 0.5, 2, 6, 10, 16, 24, or 48 h after UVB and thymine dimer positive cells in the epidermis were measured. In **(B)**, female SKH-1 mice (7–8 weeks old; 5 mice/group) were treated topically with 100 mg vehicle cream, or 24 μmol of caffeine or caffeine-SB in vehicle cream 0.5 h before UVB (180 mJ/cm^2^), and these treatments were repeated 24 h later. The area (mm^2^) and the intensity of red color in UVB-induced skin lesions were estimated 5 days after the first UVB treatment. The sunburn lesion index was calculated as the lesion area (mm^2^) multiplied by the arbitrary intensity: 0, no lesion; (1) barely detectable red lesion; (2) moderate red lesion; and (3) bright red lesion. Each value is the mean ± SE from five mice. % inhibition was calculated by comparing with the vehicle cream group (Control). Taken from Lu et al. (2007).

Since caffeine and caffeine sodium benzoate (a related, more potent inhibitor of UVB-induced skin cancer) have appreciable UV absorption between 260 and 300 nm (with a peak at ∼273 nm), we studied the effect of topical application of these compounds prior to UVB irradiation on UVB-induced thymine dimers and sunburn lesions in the epidermis of SKH-1 mice. Topical application of caffeine or caffeine sodium benzoate 0.5 h prior to UVB irradiation inhibited UVB-induced formation of thymine dimers and inhibited UVB-induced sunburn lesions, and caffeine sodium benzoate was more effective than caffeine (Figure 1) (Lu et al., 2007).

## Stimulatory Effect of Topical Caffeine or Caffeine Sodium Benzoate on UVB-Induced Apoptosis When Given Immediately after UVB in SKH-1 Mice

**Figure 2:**
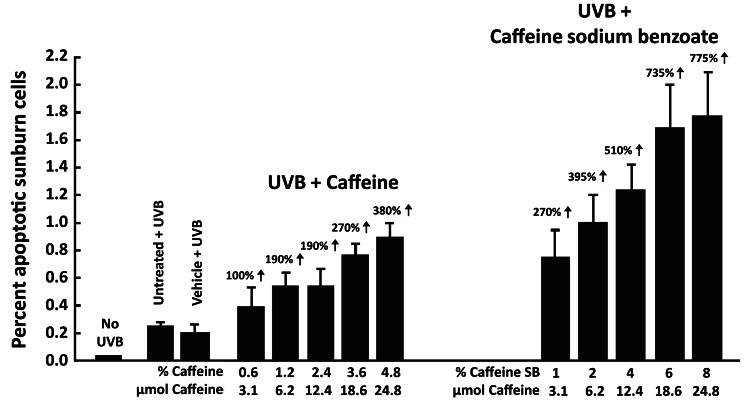
**Stimulatory effect of topical application of caffeine or caffeine sodium benzoate in a vehicle cream immediately after UVB irradiation on UVB-induced apoptosis in the epidermis of SKH-1 mice**. Female SKH-1 mice (7–8 weeks old, five per group) were treated topically once with (a) 100 mg of vehicle cream (Dermabase cream) per mouse containing 3.1, 6.2, 12.4, 18.6, or 24.8 μmol of caffeine (0.6–4.8%) or (b) 100 mg vehicle cream containing equimolar amounts of caffeine sodium benzoate (1.0–8.0%) immediately after irradiation with 30 mJ/cm^2^ of UVB. The control mice were either untreated or treated with vehicle cream alone immediately after irradiation with 30 mJ/cm^2^ of UVB. All mice were killed 6 h after UVB. Apoptotic sunburn cells in the epidermis were determined morphologically. Mice without UVB treatment had 0.01 ± 0.01% apoptotic cells in their epidermis. Each value represents the mean ± SE from five mice. % increase was calculated by comparing with “Vehicle + UVB.” Taken from Lu et al. (2007).

Since caffeine and caffeine sodium benzoate have a sunscreen effect, this was avoided by evaluating the effect of the compounds on UVB-induced apoptosis by applying them immediately after UVB irradiation. Topical application of 3.1–24.8 μmol of caffeine or caffeine sodium benzoate to SKH-1 mice immediately after irradiation with UVB (30 mJ/cm^2^) caused a dose-dependent increase in UVB-induced apoptosis (Figure 2) (Lu et al., 2007), and caffeine sodium benzoate was more effective than caffeine. Administration of caffeine or caffeine sodium benzoate in the absence of UVB was inactive (data not presented).

## Stimulatory Effect of Oral Administration of Green Tea or Caffeine on UVB-Induced Increases in p53, p21, and Apoptotic Sunburn Cells in SKH-1 Mice

**Figure 3:**
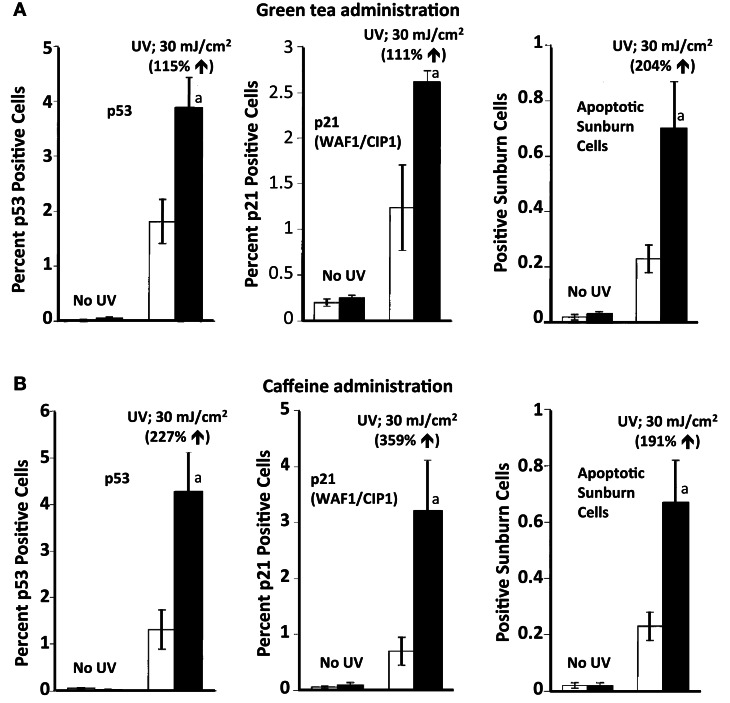
**Stimulatory effect of oral administration of green tea or caffeine on UVB-induced increases in p53 positive cells, p21 positive cells, and apoptotic sunburn cells in the epidermis of SKH-1 mice**. In **(A)**, female SKH-1 mice were treated with water (□) or green tea (6 mg of solids/ml; ■) as their sole source of drinking fluid for 2 weeks. The mice were then treated with UVB (30 mJ/cm^2^) and were killed 10 h later. In **(B)**, female SKH-1 mice were treated with water (□) or caffeine (0.44 mg/ml; ■) as their sole source of drinking fluid for 2 weeks. The mice were then treated with UVB (30 mJ/cm^2^) and were killed 10 h later. In **(A,B)**, each value represents the mean ± SE from five mice; ^*a*^*P* < 0.05. Stimulatory effect of green tea or caffeine was shown as % increase by comparing with “water + UVB.” Taken from Lu et al. (2000).

SKH-1 mice were treated orally with green tea (6 mg tea solids/ml drinking fluid) or caffeine (0.44 mg/ml in the drinking fluid) for 2 weeks prior to irradiation with UVB (30 mJ/cm^2^). Pretreatment with oral green tea or caffeine enhanced UVB-induced increases in p53 positive cells, p21 positive cells, and apoptotic sunburn cells (Figure 3) (Lu et al., 2000). Oral administration of coffee (10 mg coffee solids/ml) had a similar stimulatory effect on UVB-induced apoptosis (Conney et al., 2007). Oral administration of green tea or caffeine had no effect on p53, p21, or apoptosis in the absence of UVB irradiation indicating that these agents enhanced apoptosis only in DNA damaged epidermis but not in normal epidermis (Figure 3). To our knowledge, these studies provided the first *in vivo* demonstration of the upregulation of a tumor suppressor by a cancer preventive agent.

In normal human and mouse keratinocytes with functional p53, UVB irradiation increased the levels of p53 and p21 (Lu et al., 2000; Lei et al., 2010). However, in studies with cultured human HaCaT keratinocytes with defective p53 by Lei et al. (2010) UVB irradiation markedly decreased the level of p21, which was associated with enhanced apoptosis. Inhibition of MDM2 and GSK3β (but not inhibition of ATR/ATM by siRNAs or caffeine) prevented the UVB-induced downregulation of p21 and prevented UVB-induced apoptosis (Lei et al., 2010). It will be important to determine the *in vivo* significance of these studies by doing studies in mice with UVB-induced p53 mutations in their epidermal cells. Studies of p21 expression and apoptosis in p53 knockout mice will also help define the relationship of UVB and caffeine to p21 in this particular situation.

## Stimulatory Effect of Topical Application of Caffeine on UVB-Induced Apoptosis in the Epidermis of p53 Knockout Mice

**Figure 4:**
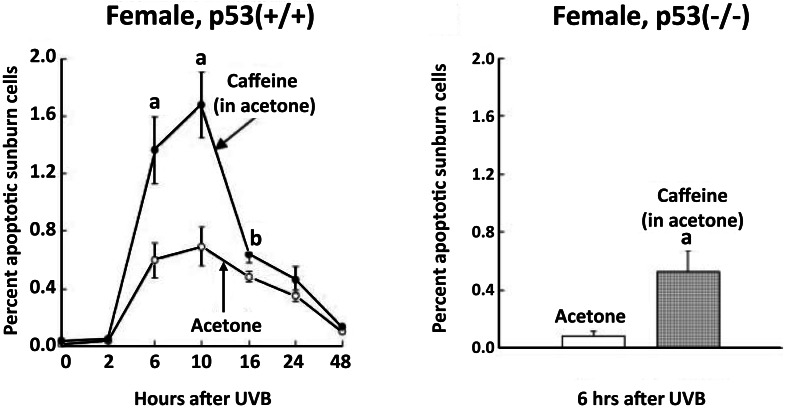
**Stimulatory effect of topical application of caffeine on UVB-induced apoptosis in the epidermis of p53 knockout mice**. Female p53(+/+) and p53(-/-) mice (7–8 weeks old, five mice for each time interval) were treated with 100 μl of acetone or caffeine (1.2 mg; 6.2 μmol) in 100 μl of acetone immediately after UVB (60 mJ/cm^2^) and 0.5 and 2 h later. The animals were killed at the indicated times. Morphologically distinct apoptotic sunburn cells were counted. Each value represents the mean ± SE. Statistically significant differences after UVB exposure were observed for the percentage of apoptotic sunburn cells between the acetone-treated control animals and the caffeine-treated animals (*^*a*^P* < 0.01; *^*b*^P* < 0.05). Taken from Lu et al. (2004).

Although UVB-induced apoptosis in the epidermis was markedly decreased in p53 knockout mice, topical application of caffeine immediately after UVB irradiation in these knockout mice markedly stimulated UVB-induced apoptosis by a p53-independent effect (Figure 4) (Lu et al., 2004).

## Inhibitory Effect of Caffeine or siRNA for ATR on the ATR/Chk1 Pathway Leads to Enhanced Apoptosis in Primary Human Keratinocytes

**Figure 5:**
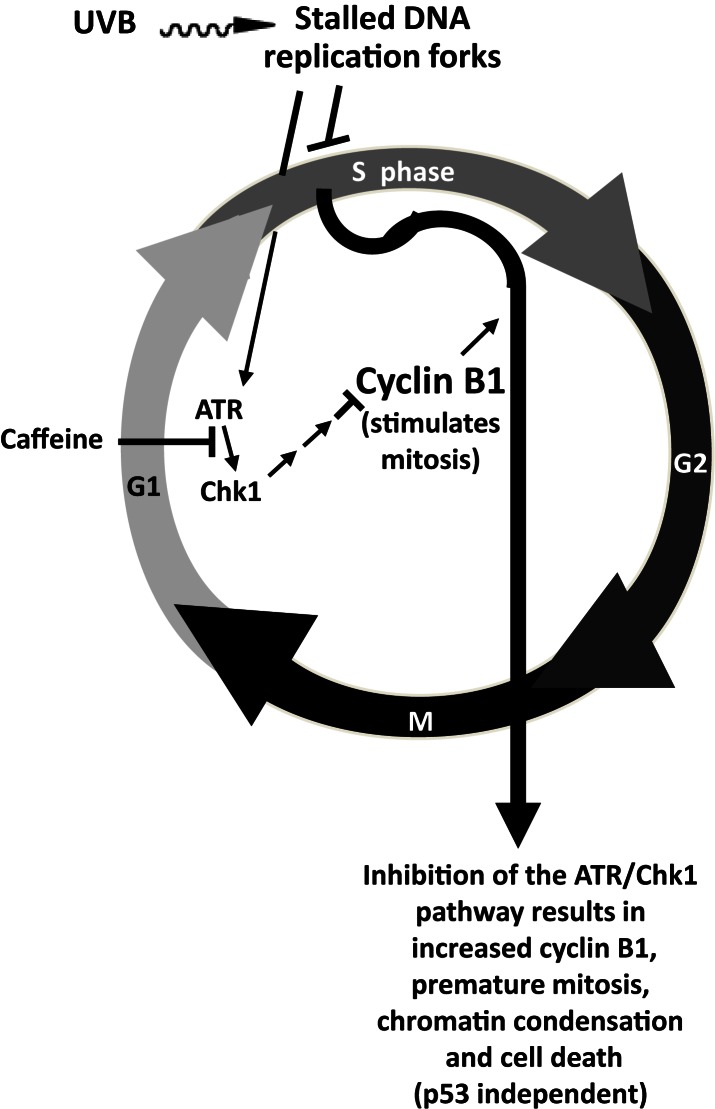
**Proposed effects of UVB and caffeine on the ATR/Chk1/cyclin B1 pathway and premature mitosis**. The signal that DNA synthesis is not complete (and that chromatin condensation and mitosis should not start) after UVB irradiation is sent by ATR after it is recruited to stalled replication forks that have been coated by replication protein A. ATR-mediated phosphorylation of Chk1 after UVB irradiation results in a decreased level and function of cyclin B1, which delays mitotic entry and prevents premature chromatin condensation and mitosis. Inhibition of the ATR/Chk1 pathway in UVB-treated cells should result in a premature increase in cyclin B1 and premature chromatin condensation, mitosis, and cell death, probably by mitotic catastrophe followed by apoptosis, as suggested by Brown and Attardi (2005). Adapted with permission from Nghiem et al. (2001) Taken from Lu et al. (2008).

A schematic figure relating the effects of ATR-mediated phosphorylation of Chk1 on premature upregulation of cyclin B1 and cell death is shown in Figure 5 (Lu et al., 2008).

**Figure 6:**
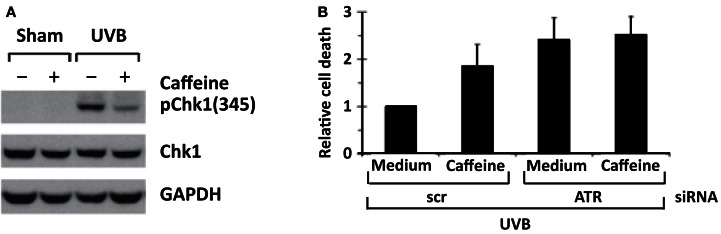
**ATR siRNA mimics caffeine by inhibiting Chk1 phosphorylation and augmenting apoptosis after UV treatment in primary human keratinocytes**. In **(A)**, human keratinocytes (HKC) were treated with vehicle (medium) or 2 mM of caffeine 30 min before 75 mJ/cm^2^ of UVB irradiation (the caffeine was removed before irradiation and returned to cell culture medium after UV in order to avoid UV-absorption effect of caffeine). Cells were harvested 2 h after UVB irradiation. In **(B)**, HKC were electroporated with siRNA against ATR or a non-targeting/scrambled control (scr). Forty-eight hours later, cells were exposed to 75 mJ/cm^2^ of UVB, and the cells were harvested 24 h later. Relative cell death was calculated by comparing percentage of sub-2N DNA content in scr + caffeine/UVB-, siRNA for ATR + medium/UVB-, or siRNA for ATR + caffeine/UVB-treated cells with that in scr + medium/UVB-treated cells in each experiment. Average of relative cell death is shown (*n* = 3). Error bar, standard error of the mean. Taken from Heffernan et al. (2009).

Treatment of primary human keratinocytes with caffeine inhibited UVB-induced increase in ATR-mediated formation of p-Chk1 (Ser 345), and apoptosis was increased (Figure 6) (Heffernan et al., 2009). Genetic inhibition of ATR by siRNA for ATR also enhanced UVB-induced apoptosis, which was not increased further by the addition of caffeine (Figure 6) (Heffernan et al., 2009). These results suggest that caffeine enhances UVB-induced apoptosis in primary human keratinocytes predominantly by inhibiting ATR-mediated phosphorylation of Chk1. Our cell culture studies are in agreement with *in vivo* studies presented later in this review indicating that transgenic mice with diminished ATR function have decreased UVB-induced tumorigenesis when compared with littermate controls with normal ATR.

In studies with cultured human HaCaT keratinocytes with defective p53 by Han et al. (2011), treatment with caffeine enhanced UVB-induced apoptosis, inhibited UVB-induced phosphorylation of Chk1 as well as the phosphorylation of AKT and the upregulation of COX-2. In these cells, siRNA inhibition of ATR did not have a significant effect on UVB-induced apoptosis (Han et al., 2011), which differed from our data obtained from studies with primary human keratinocytes (Figure 6) (Heffernan et al., 2009). The reasons for differences in the effects of siRNA for ATR on UVB-induced apoptosis between the two cell lines may be related to different properties of the various cells lines or to the use of different incubation conditions for the two studies. The lack of effect of siRNA for ATR on UVB-induced apoptosis observed by Han and his colleagues may have resulted from incubation conditions whereby maximum apoptosis already occurred prior to the addition of siRNA for ATR. The *in vivo* importance of decreasing ATR function for inhibition of UVB carcinogenesis was demonstrated by showing that decreasing ATR function in transgenic mice inhibited UVB-induced carcinogenesis (see Figure 11). Additional studies are needed to determine the *in vivo* effects of caffeine administration on UVB-induced phosphorylation of AKT and UVB-induced upregulation of COX-2 in the epidermis of p53 proficient and p53 deficient mice.

## Caffeine Inhibition of UVB-Induced Increase in Phospho-Chk1 (Ser 345) Associated with Premature Increase in Cyclin B1 and Increased Apoptosis in the Epidermis of SKH-1 Mice

**Figure 7:**
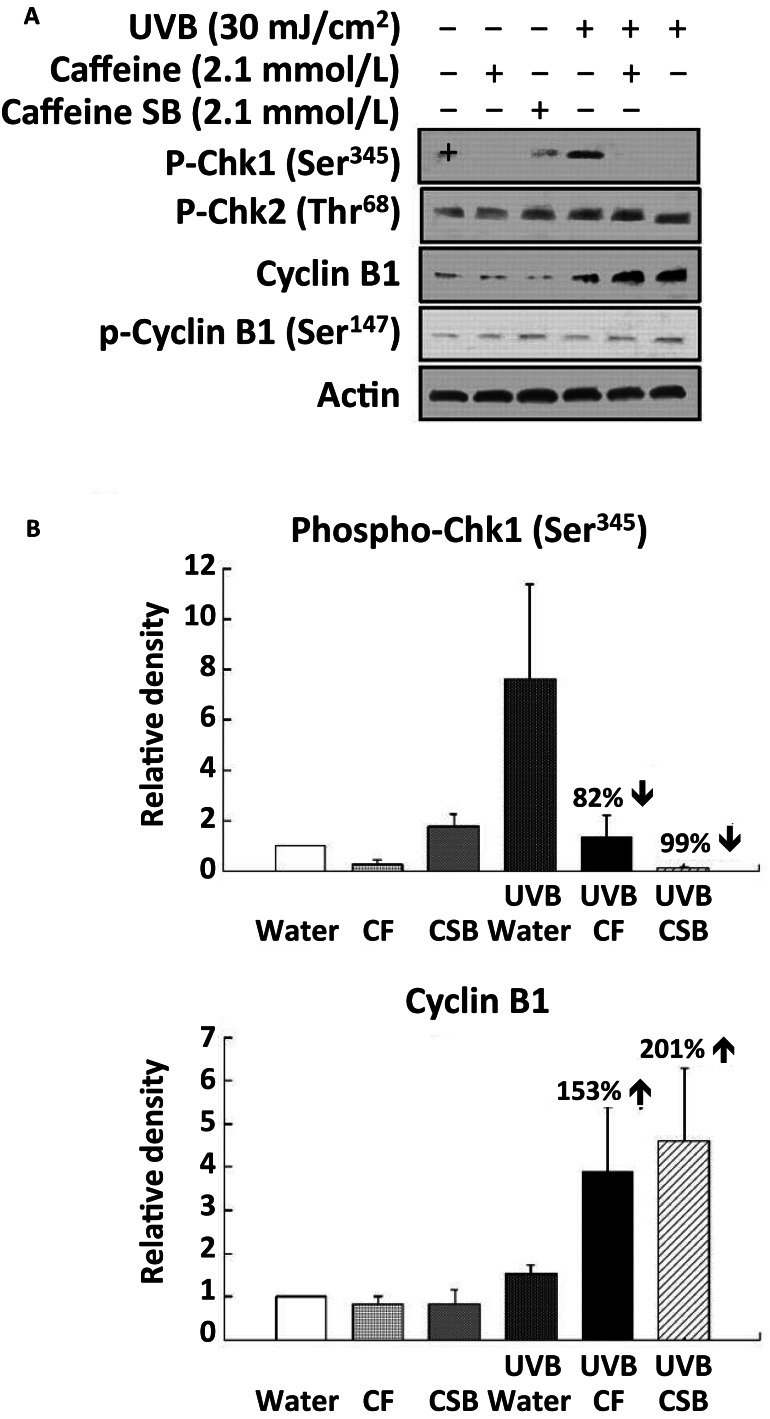
**Effects of oral caffeine (CF) or caffeine sodium benzoate on UVB-induced changes in phospho-Chk1 (Ser 345) and cyclin B1 in the epidermis of SKH-1 mice: western blot studies**. Female SKH-1 mice were treated p.o. with caffeine (CF; 0.4 mg/ml, 2.1 mmol/l) or caffeine sodium benzoate (CSB, caffeine-SB; 2.1 mmol/l) as their sole source of drinking fluid for 1 week. The animals were then treated with UVB (30 mJ/cm^2^) and killed 6 h later. In **(A)**, phospho-Chk1 (Ser345), phospho-Chk2 (Thr68), cyclin B1, and phospho-cyclin B1 (Ser147) were determined by Western blot. In **(B)**, the average densitometry results from three independent experiments are shown. Taken fromLu et al. (2008).

**Figure 8:**
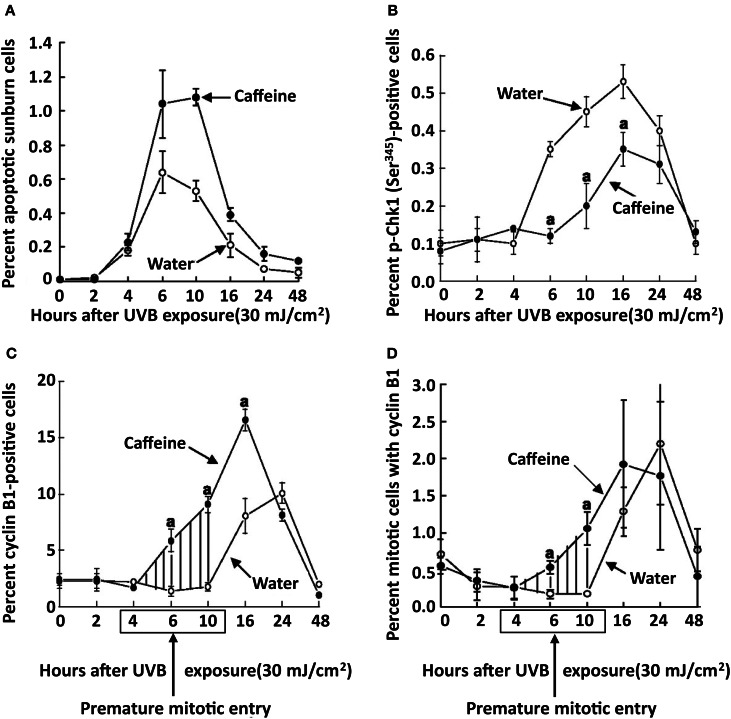
**Time course for the effects of oral caffeine on UVB-induced changes in the percentage of apoptotic sunburn cells, phospho-Chk1 (Ser 345) positive cells, cyclin B1 positive cells, and mitotic cells with cyclin B1 in the epidermis of SKH-1 mice**. Female SKH-1 mice were treated p.o. with caffeine (0.4 mg/ml, 2.1 mmol/l) in the drinking water for 2 week. Control mice received only water. The animals were then irradiated with UVB (30 mJ/cm^2^) and killed at the indicated times. The percentage of phospho-Chk1 (Ser345) and cyclin B1-positive cells were determined immunohistochemically. Cells with both mitosis (measured by morphology) and cyclin B1 (measured by immunohistochemistry) were also determined. Points, mean of five mice; bars, SE. Shaded areas, premature mitotic entry by epidermal cells from caffeine-treated mice. ^*a*^*P* < 0.01. Taken from Lu et al. (2008).

Treatment of SKH-1 mice with caffeine or caffeine sodium benzoate (2.1 mmol/l as the drinking fluid) for 1 week inhibited the UVB-induced increase in p-Chk1 (Ser 345) by 82 and 99%, respectively at 6 h post-UVB, and cyclin B1 was increased 153 and 201%, respectively at 6 h post-UVB (Figure 7) (Lu et al., 2008). The time course for the UVB-induced increase in p-Chk1 (Ser 345), and the effect of caffeine to abrogate the UVB-induced increase in p-Chk1 (Ser 345) and the decrease in cyclin B1 is shown in Figure 8 (Lu et al., 2008). Caffeine-induced abrogation of the UVB-induced decrease in cyclin B1 is associated with premature mitosis and cell death (Figure 8).

## Inhibitory Effect of Caffeine on Tumor Formation in UVB-Pretreated High-Risk Mice

Treatment of SKH-1 mice with UVB (30 mJ/cm^2^) twice a week for 20 weeks resulted in mice without tumors but with epidermal hyperplasia and a high risk of developing tumors over the next several months in the absence of further treatment with UVB (“high-risk mice”) (Lou et al., 1999). Treatment of these UVB-pretreated “high-risk mice” with oral caffeine (Lou et al., 1999) or with topical applications of caffeine (Lu et al., 2002) inhibited tumor formation. The inhibitory effect of topical applications of caffeine on tumor formation in high-risk mice is shown in Table 2. Treatment of high-risk mice with topical applications of caffeine (6.2 μmol) once a day, five times per week for 18 weeks inhibited the percent of mice with squamous cell carcinomas by 64% and the number of carcinomas/mouse by 72%. Administration of caffeine increased apoptosis in the tumors but not in areas of the epidermis away from tumors indicating selectivity for caffeine action on tumors but not in areas away from tumors (Table 3) (Lu et al., 2002).

**Table 2:** **Inhibitory effect of topical applications of caffeine on tumor formation in UVB-pretreated high-risk mice**.

Treatment	Keratoacanthomas		Squamous cell carcinomas	
	Percent of mice with tumors	Tumors per mouse	Percent of mice with tumors	Tumors per mouse
Acetone	82	7.07 ± 1.27	64	1.18 ± 0.25
Caffeine	77 (6% ↓)	3.93 ± 0.74^*a*^ (44% ↓)	23^*a*^ (64% ↓)	0.33 ± 0.12^*a*^ (72% ↓)

*High-risk UVB-pretreated SKH-1 mice (30/group) were treated topically with 100 μl acetone or caffeine (6.2 μmol) in 100 μl acetone once daily 5 days a week for 18 weeks. The numbers in parentheses represent percent inhibition. Each value represents the mean ± SE ^a^p < 0.01. Taken from Lu et al. (2002)*.

**Table 3:** **Topical applications of caffeine to UVB-pretreated high-risk mice stimulates apoptosis in the tumors**.

Treatment	No. of tumors examined	Percent caspase 3 positive cells	Percent increase
**NON-TUMOR AREAS**			
Control	-	0.159 ± 0.015	-
Caffeine	-	0.165 ± 0.027	4
**KERATOACANTHOMAS**			
Control	198	0.229 ± 0.017	-
Caffeine	118	0.430 ± 0.034^*a*^	88
**CARCINOMAS**			
Control	33	0.196 ± 0.022	-
Caffeine	10	0.376 ± 0.056^*a*^	92

*High-risk UVB-pretreated mice (30/group) were treated topically with acetone (100 μl) or with caffeine (6.2 μmol) in 100 μl acetone once daily 5 days a week for 18 weeks as shown in Table 2. Each value for the percent of caspase 3 positive cells represents the mean ± SE ^a^p < 0.01. Taken from Lu et al. (2002)*.

## Effect of Caffeine on the ATR/Chk1 Pathway in UVB-Induced Tumors

**Figure 9:**
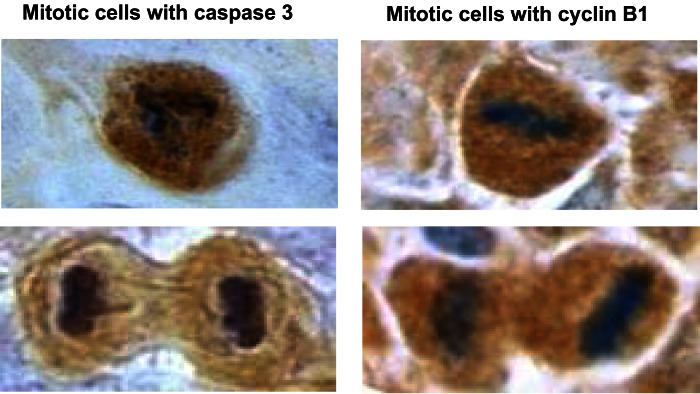
**Illustration of mitotic cells with caspase 3 or cyclin B1 staining**. This figure shows representative mitotic cells with caspase 3 (left) or mitotic cells with cyclin B1 (right) positive staining (microscope magnification = 1000-fold). The tumor sample was from a UVB-pretreated high-risk mouse treated with caffeine for 18 weeks. Similar images were obtained in tumors from control mice. Taken from Lu et al. (2011).

Topical applications of caffeine to UVB-pretreated high-risk mice inhibited carcinogenesis as described in Table 2 and enhanced apoptosis in the tumors (but not in non-tumor areas) as described in Table 3. Immunohistochemistry revealed islands of phospho-Chk1 (Ser 317) in the tumors but not in areas away from the tumors, and caffeine administration decreased the number of positively stained islands and also decreased the intensity of staining (Lu et al., 2011). We also observed that some mitotic cells in the tumors were positive for cyclin B1 staining and for caspase 3 (active form) staining. Representative mitotic tumor cells with cyclin B1 or apoptosis (caspase 3, active form) staining are shown in Figure 9 (Lu et al., 2011).

**Figure 10:**
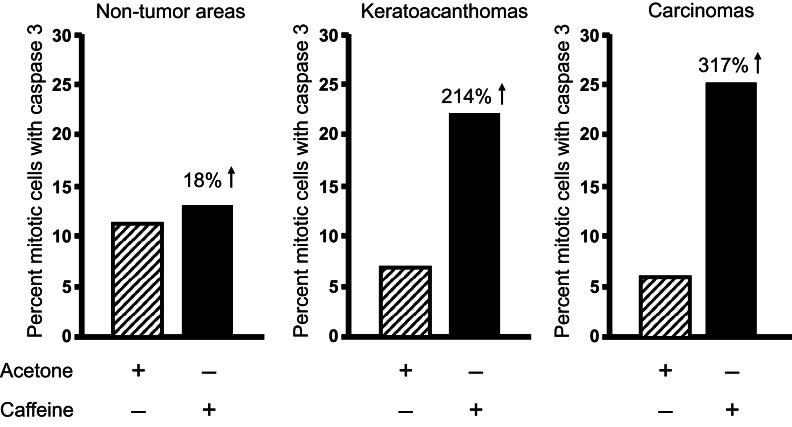
**Topical applications of caffeine to UVB-pretreated high-risk mice increase the percentage of mitotic cells with caspase 3 in tumors**. High-risk UVB-pretreated mice were treated topically with caffeine for 18 weeks as described in Table 2. Mitotic cells with caspase 3 were measured. Taken from Lu et al. (2011).

Topical caffeine increased the percentage of mitotic tumor cells with cyclin B1 by 70% (Lu et al., 2011), and the percentage of mitotic keratoacanthoma and squamous cell carcinoma cells with caspase 3 (active form) was increased by 214 and 317%, respectively (Figure 10) (Lu et al., 2011). Interestingly, caffeine administration did not increase the percentage of mitotic cells with caspase 3 in non-tumor areas of the epidermis in tumor-bearing mice.

## Effect of Genetic Inhibition of ATR/Chk1 Pathway on UVB-Induced Carcinogenesis in Transgenic Mice

**Figure 11:**
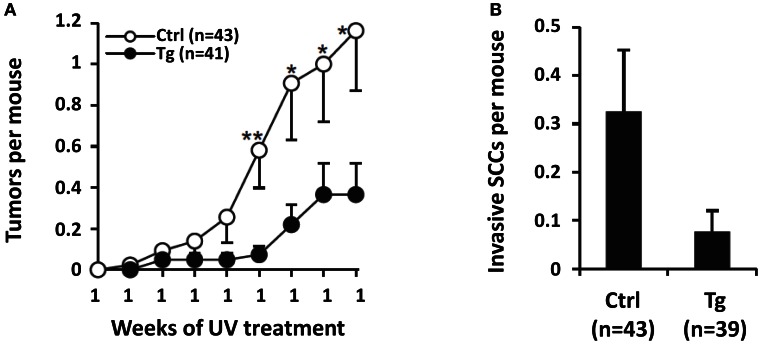
**Effect of genetic inhibition of ATR on UVB-induced carcinogenesis**. Transgenic mice with decreased ATR function in the skin were crossed into a UV-sensitive Xpc^−*/*−^ background. These transgenic mice (Tg) and their transgenic-negative littermate controls with functional ATR (Ctrl) were treated with UVB for 23 weeks. In **(A)**, it is shown that ATR-kd transgene suppresses UV-induced tumor development. Mean number of tumors per mouse is shown up to 19 week, the point when some mice with advanced tumors were killed and the cohort was no longer complete (Error bars = SEM). Statistical significance in mean number of tumors per mouse between the groups was as shown at the indicated time points: *P ≤ 0.05, **P < 0.01. In **(B)**, transgenic mice with diminished ATR function (Tg) had fourfold fewer clinically defined invasive SCCs than transgene-negative littermate controls (Ctrl) did, although this result had only borderline statistical significance (*P* = 0.071). Mean number of invasive SCCs per mouse after 23 week of UV treatment is shown (Error bars = SEM). Taken from Kawasumi et al. (2011).

To determine the effect of genetic inhibition of ATR on UVB-induced carcinogenesis, we generated transgenic mice with diminished ATR function in skin and crossed them into a UV-sensitive Xpc^−*/*−^ background. Unlike caffeine, this genetic approach was selective for ATR function and had no effect on ATM activation by DNA damage. Primary keratinocytes from these mice had diminished UV-induced Chk1 phosphorylation and a twofold increase in apoptosis after UV exposure. With chronic UV treatment, transgenic mice remained tumor-free for significantly longer and had 69% fewer tumors at the end of observation of the full cohort than in littermate controls (Figure 11) (Kawasumi et al., 2011).

## Oral Caffeine Increases Locomotor Activity and Decreases Tissue Fat

Since oral administration of caffeine increased locomotor activity and decreased tissue fat in SKH-1 mice (Lu et al., 2001; Michna et al., 2003), we evaluated the effect of voluntary running wheel exercise and removal of the parametrial fat pads on UVB-induced apoptosis and UVB-induced skin cancer (Lu et al., 2006, 2012; Michna et al., 2006). Both running wheel exercise and removal of the parametrial fat pads had the same effects as oral caffeine administration – stimulation of UVB-induced apoptosis, inhibition of UVB-induced carcinogenesis, and enhanced apoptosis in the tumors of SKH-1 mice treated chronically with UVB (Lu et al., 2006, 2012; Michna et al., 2006). The results suggest that oral administration of caffeine may inhibit UVB-induced carcinogenesis in part by increasing locomotor activity and by decreasing tissue fat.

## Conclusion

Animal data indicate that caffeine administration inhibits UVB-induced carcinogenesis by functioning as a sunscreen as well as by enhancing UVB-induced apoptosis and apoptosis in UVB-induced tumors. The stimulatory effect of caffeine on UVB-induced apoptosis occurs by p53-dependent and p53-independent mechanisms. Inhibition of the ATR/Chk1 pathway by caffeine is a major contributor to caffeine inhibition of UVB-induced carcinogenesis. The inhibitory effects of caffeine on UVB-induced carcinogenesis in animal studies described here are paralleled by an inhibitory effect of regular but not decaffeinated coffee on non-melanoma skin cancer in humans (Jacobson et al., 1986; Abel et al., 2007; Song et al., 2012).

## Conflict of Interest Statement

The authors declare that the research was conducted in the absence of any commercial or financial relationships that could be construed as a potential conflict of interest.
